# Identification and antimicrobial susceptibility profile of bacteria isolated from primary endodontic infections

**DOI:** 10.1590/1807-3107bor-2024.vol38.0024

**Published:** 2024-04-05

**Authors:** Lorena Souza Santos MARIANO, Rafael NAKAMURA-SILVA, Luciana Martins Domingues de MACEDO, Mariana de OLIVEIRA-SILVA, Rafael da Silva GOULART, Marsileni PELISSON, Eliana Carolina VESPERO, Yara Teresinha Correa SILVA-SOUSA, André PITONDO-SILVA

**Affiliations:** (a) Universidade de Ribeirão Preto, Postgraduate Program in Dentistry, Ribeirão Preto, SP, Brazil.; (b) Universidade de Ribeirão Preto, Postgraduate Program in Environmental Technology, Universidade de Ribeirão Preto, Ribeirão Preto, Brazil.; (c) Universidade Estadual de Londrina, Department of Pathology, Clinical and Toxicological Analysis, Londrina, PR, Brazil.

**Keywords:** Drug Resistance, Microbial, Bacterial Infections, Endodontics, Dental Pulp Cavity

## Abstract

This study aimed to identify and characterize the antimicrobial susceptibility profile of bacteria found in primary endodontic infections in the teeth of patients treated at the Dental Clinic of the University of Ribeirão Preto, São Paulo, Brazil. From September to December 2019, samples were obtained from 21 patients with primary endodontic infections. The collections were carried out in triplicate using paper cones placed close to the total length of the root canal. Bacterial isolation was performed in Brain Heart Infusion agar, Blood agar, and other selective culture media cultured at 37°C for up to 48 h under aerobiosis and microaerophilic conditions. The bacterial species were identified using the Vitek 2 automated system. The disk diffusion method on agar Müeller–Hinton was used to assess antimicrobial susceptibility with the recommended antimicrobials for each identified bacterial species. A total of 49 antibiotics were evaluated. Fifteen of the 21 samples collected showed bacterial growth, and 17 bacterial isolates were found. There were 10 different bacterial species identified: *Enterococcus faecalis* (four isolates), Streptococcus mitis/oralis (three isolates), Streptococcus anginosus (three isolates) being the most common, followed by *Staphylococcus epidermidis, Enterococcus faecium, Streptococcus constellatus, Streptococcus alactolyticus, Enterobacter cloacae, Klebsiella variicola*, and *Providencia rettgeri* (one isolate of each species). The analysis demonstrated significant susceptibility to most of the tested antibiotics. However, some *Enterococcus* isolates resisted the antibiotic’s erythromycin, ciprofloxacin, and tetracycline. *A Staphylococcus* epidermidis isolate was characterized as multidrug-resistant. Five Streptococcus isolates were non-susceptible to all antibiotics tested.

## Introduction

The dentin–pulp complex is pathogen-free and sterile when in a normal state. The tooth lining materials, cement and enamel, act as a barrier to prevent microorganisms from penetrating the pulp tissue. The pulp is susceptible to pathogens entering the oral cavity through dentinal tubules or pulp exposure when one of these structures is compromised by conditions such as caries, periodontal disorders, or trauma. This contamination of pulp can lead to necrosis.^1,2^

Theoretically, any microorganism that enters the root canal system (RCS) can cause endodontic infection. Therefore, oral microbiota members and foreign microbes penetrating the oral cavity are considered pathogens. However, some bacteria have greater adaptability to adverse conditions and virulence factors, allowing them to colonize the teeth and cause infections more frequently.^1,3,4^

Numerous bacterial species, such as those of the genus Fusobacterium, Prevotella, Porphyromonas, Prevotella, Parvimonas, Propionibacterium, Streptococcus, Treponema, and Olsenella, have been associated with primary endodontic infections.^4,5^ However, it is estimated that approximately half of the bacterial species found in infected root canals of teeth with primary apical periodontitis are still not cultivable, which makes their identification extremely challenging. The microbiological culture of these species is essential to evaluate their characteristics, including the profile of pathogenicity and susceptibility to antimicrobials.^4,6^

All facets of healthcare, including dentistry, underline the vital significance of antibiotic prescriptions and the ensuing risk of developing antimicrobial resistance. Estimates indicate that around 10% of antibiotic prescriptions are associated with dental practice, and, in some situations, their use is not necessarily based on accurate indications. Based on focused oral sepsis theory, antibiotics are often prescribed during dental treatments. According to this notion, there may be a risk of bacteremia associated with infections or surgical procedures in the oral region. However, the empirical prescription of antibiotics without first identifying, cultivating, and testing the in vitro susceptibility of the microorganisms implicated is crucial in dentistry.^7^ These aspects highlight the importance of the rational use of antibiotic prescriptions in dentistry to reduce the dissemination of multidrug-resistant bacteria.^8,9^ The oral microbiota is a reservoir for several antibiotic-resistance genes, including the most commonly used antibiotic classes, such as beta-lactams, tetracycline, and macrolides.^10^ This is quite concerning because these antibiotics are commonly prescribed to treat oral and endodontic infections.^3,6^ It suggests that antibiotics in endodontic infections should be used only as an adjunct to treating these infections. When properly indicated, it is crucial for endodontic therapy’s success.^11^

Antibiotics are not required to treat the majority of endodontic infections. Endodontic infections are unresponsive to systemic antibiotic therapy. Because necrotic pulps lack blood circulation, they are unable to eliminate microorganisms present in the RCS.^11^ When antibiotic therapy is required to treat endodontic infections, the antibiotic is typically selected based on clinical reports previously published in the literature and, in rare cases, antimicrobial susceptibility tests. Because of their effectiveness against most bacteria that cause endodontic infections and their low adverse effects, β-lactam antibiotics, particularly those from the penicillin family, were the first choice. When penicillin fails, β-lactamase inhibitors, such as clavulanic acid, are combined with amoxicillin.^12^

Antibiotic-resistant bacterial strains have emerged in all areas of health, owing primarily to their abusive and incorrect use. The rational prescription of antimicrobials should be based on the bacteria resistance profile, the patient’s specific characteristics, and the clinical procedure performed.^12^ Dentists must understand the characteristics of the infection to be treated; this enables professionals to use antibiotics wisely and, when necessary, to prescribe antibiotics with an appropriate spectrum of action, dose, frequency, and duration of treatment.^13^ This approach may help reduce the spread of antimicrobial bacterial resistance, one of today’s most pressing scientific concerns.

Traditional biochemical methods have been used to detect microbial species that are difficult to identify using automated methods. Several automated identification systems covering various biochemical tests are available on the market, with the most popular being the Vitek 2 system (Biomérieux, France). The Vitek system is suitable for identifying microorganisms isolated in routine clinical microbiology laboratories because the specificity analysis demonstrated a performance greater than 90%, which is required for commercial clinical microbiology systems. The Vitek 2 system uses bacterial metabolism to identify bacteria by evaluating a series of miniaturized biochemical tests on specific cards containing approximately 40 biochemical tests.^14,15,16^

This study aims to identify and characterize the antimicrobial susceptibility profile of bacteria found in primary endodontic infections in the teeth of patients treated at the University of Ribeirão Preto Dental Clinic in São Paulo, Brazil.

## Methodology

### Selection of patients and clinical cases

This study included 21 patients with primary endodontic infections treated at the Dental Clinic of the University of Ribeirão Preto in São Paulo, Brazil. According to the inclusion and exclusion criteria, the sample corresponds to the total number of endodontic consultations performed from September to December 2019. Patients in the study had not used antibiotics in the 3 months before collection and had not previously received endodontic treatment. Cases of gingivitis and dental mobility were excluded, as were cases of the impossibility of absolute isolation of the tooth, the impossibility of inserting the paper cones into the root canals up to the vicinity of the apparent length of the tooth observed on radiography, and cases of gingivitis and dental mobility.

### Sample collection

Antisepsis of the patient’s face with 0.2% chlorhexidine was performed before endodontic surgery, and local anesthesia was administered in the region of the involved tooth. Before being isolated completely, the tooth was prophylaxed with pumice and Robson’s brush. After that, the surgical field was disinfected for 30 s with sterile swabs dampened with 2.5% sodium hypochlorite (NaClO). The coronary opening was performed in high rotation with a spherical diamond tip and sterile saline refrigeration. Using sterile paper cones compatible with the canal’s anatomical diameter, the biological material was collected immediately after the coronary opening and before the canal’s toxic septic content was neutralized.^17^

To allow biological material absorption, paper cones were introduced close to the total length of the root canal as determined by diagnostic radiography, touching the internal walls and remaining in a static position for 1 min. When the RCS was dry, the cones were moistened with sterile saline solution (0.9% NaClO) before collection to ensure the collection of a viable sample, and multiple cones were collected to increase the chances of containing microorganisms. This process was repeated three times to obtain a more diverse microbiological sampling, totaling three paper cones collected for each sample. The same endodontic specialist performed all collections.

The cones were immediately removed from the root canal and placed in sterile 2-mL cryotubes containing 1.7 mL of brain heart infusion (BHI) broth (Acumedia, USA). The tubes were homogenized, refrigerated, and transported to the microbiology laboratory within 24 h for bacterial culture and isolation.

### Bacterial isolation and identification

The cones were immersed in a BHI liquid medium for 24 h before homogenization and incubated under aerobic and microaerophilic conditions (5% CO_2_) at 37°C. Following this period, 10 μL of these cultures were inoculated in Petri dishes containing BHI agar (Acumedia, USA), Blood agar (Laborclin, Brazil), and other selective culture media, using a sterile inoculation loop (Sigma-Aldrich, USA).^18^ They were then incubated separately for up to 48 h at 37°C in aerobiosis and microaerophilic (5% CO_2_) conditions, aiming at the growth of obligate aerobes, facultative anaerobes, aerotolerant anaerobes, and microaerophiles bacterial species. After the Gram stain, the bacterial colonies were visually examined for macroscopic and microscopic morphology.^18^

Individual colonies with various bacterial morphologies were isolated and identified on BHI agar. The bacteria were homogenized and cryopreserved in BHI broth with 15% glycerol at −80°C. When viable bacterial cultures were frozen, glycerol was used as a cryoprotectant.

According to the manufacturer’s instructions, the bacterial species were identified using the automated biochemical identification system Vitek 2 (Biomérieux, France).

### Antimicrobial susceptibility testing

The disk diffusion method on agar Müeller–Hinton (Oxoid), as recommended by the Clinical Laboratory Standards Institute (CLSI), was used to test antimicrobial susceptibility with the recommended antimicrobials and concentrations for each identified bacterial species.^19^ Therefore, groups of different antimicrobial disks were tested for each species.

Forty-nine different antibiotic disks (Oxoid, UK) were tested, which may vary according to each bacterial species found, namely, amoxicillin-clavulanate, amikacin, ampicillin-sulbactam, ampicillin, aztreonam, azithromycin, ceftazidime, cefaclor, ceftaroline, cefoxitin, cefazolin, cephalothin, ciprofloxacin, clarithromycin, clindamycin, chloramphenicol, cefepime, ceftriaxone, cefuroxime, cefotaxime, doripenem, doxycycline, ertapenem, erythromycin, streptomycin, fosfomycin, gentamicin, imipenem, levofloxacin, linezolid, lomefloxacin, minocycline, moxifloxacin, meropenem, nalidixic acid, nitrofurantoin, norfloxacin, ofloxacin, oxacillin, penicillin, piperacillin-tazobactam, rifampicin, sulphonamide, trimethoprim-sulfamethoxazole, ticarcillin-clavulanate, tetracycline, tobramycin, trimethoprim, vancomycin.

Bacterial isolates with intermediate resistance were deemed resistant to the antibiotics tested. Klebsiella pneumoniae ATCC 700603, Escherichia coli ATCC 25922, *Enterobacter cloacae* ATCC 13047, *Staphylococcus aureus* ATCC 25923, *Enterococcus faecalis* ATCC 4083, and *Streptococcus mutans* ATCC 25175 were used as quality control bacterial strains in this study.

## Results

### Bacterial isolates

Fifteen of the 21 samples collected from different patients’ teeth with primary endodontic infections showed bacterial growth, whereas six samples did not. There were 17 bacterial isolates found among the 15 positive cultures. Samples E13 and E16 each grew two bacterial species (Table).

**Table:** Characteristics of the cases included in this study.

Sample	Species	Gram	Patient age	Tooth	Types and region of teeth	Tooth condition	Symptomatology	Radiological aspect	Clinical aspect
E1	*Streptococcus mitis/oralis*	+	31	15	Upper premolar	Decayed	Symptomatic	Periapical lesion	Fistula
E2	*Streptococcus anginosus*	+	7	11	Upper central incisor	Decayed	Symptomatic	Periapical lesion	-
E3	*Streptococcus anginosus*	+	62	34	Lower premolar	Decayed	Asymptomatic	-	-
E5	*Enterococcus faecalis*	+	23	11	Upper central incisor	Decayed	Asymptomatic	-	-
E10	*Enterococcus faecalis*	+	55	44	Lower premolar	Restored	Symptomatic	Periapical lesion	-
E11	*Streptococcus anginosus*	+	42	24	Upper premolar	Decayed	Symptomatic	Periapical lesion	-
E12	*Enterococcus faecalis*	+	51	11	Upper central incisor	Decayed	Symptomatic	-	-
E13A	*Streptococcus mitis/oralis*	+	56	21	Upper central incisor	Decayed	Symptomatic	Periapical lesion	Fracture
E13B	*Streptococcus constellatus*	+	56	21	Upper central incisor	Decayed	Symptomatic	Periapical lesion	Fracture
E14	*Streptococcus alactolyticus*	+	19	11	Upper central incisor	Decayed	Asymptomatic	Periapical lesion	Fistula
E15	*Streptococcus mitis/oralis*	+	19	12	Upper side incisor	Decayed	Asymptomatic	Periapical lesion	Fistula
E16A	*Enterococcus faecalis*	+	41	36	Upper molar	Restored	Symptomatic	-	-
E16B	*Enterobacter cloacae*	-	41	36	Upper molar	Restored	Symptomatic	-	-
E17	*Enterococcus faecium*	+	51	11	Upper Central Incisor	Decayed	Symptomatic	-	-
E18	*Klebsiella variicola*	-	27	21	Upper Central Incisor	Decayed	Symptomatic	Periapical lesion	Fistula
E20	*Providencia rettgeri*	-	45	13	Upper Canine	Restored	Symptomatic	-	-
E21	*Staphylococcus epidermidis*	+	32	15	Upper Premolar	Decayed	Asymptomatic	Periapical lesion	-

Ten different bacterial species were identified among the 17 bacterial isolates, with *E. faecalis* (four isolates), Streptococcus mitis/oralis (three isolates), *Streptococcus anginosus* (three isolates) being the most common, followed by *Staphylococcus epidermidis, Enterococcus* faecium, Streptococcus constellatus, Streptococcus alactolyticus, E. cloacae, *Klebsiella variicola*, and *Providencia rettgeri* (one isolate of each species, Table 1). Therefore, most of the bacterial isolates (82%) found are Gram-positive, with only three (18%) being Gram-negative (Table).

### Antimicrobial susceptibility profile

Bacterial isolates were found to be susceptible to the majority of antibiotics tested. For *E. faecalis* and *E. faecium*, most isolates were susceptible to the antibiotics tested. Some isolates, however, were resistant to erythromycin (all isolates except 10), ciprofloxacin (isolates E10, E12, and E16A), tetracycline (isolates E5, E12, and E16A), penicillin (isolates E16A and E17), chloramphenicol (isolate E12), doxycycline, and minocycline (isolates E12 and E16A).

The *Streptococcus* spp. isolates were susceptible to most antibiotics tested. All antibiotics were effective against five isolates (E1, E2, E3, E13B, and E15). Isolate 11 was resistant to azithromycin and clarithromycin, isolate E13A was resistant to azithromycin, erythromycin, clarithromycin, cefepime, ceftriaxone, cefotaxime, and clindamycin, and isolate E14 was resistant to clindamycin and tetracycline (Figure).

**Figure:**
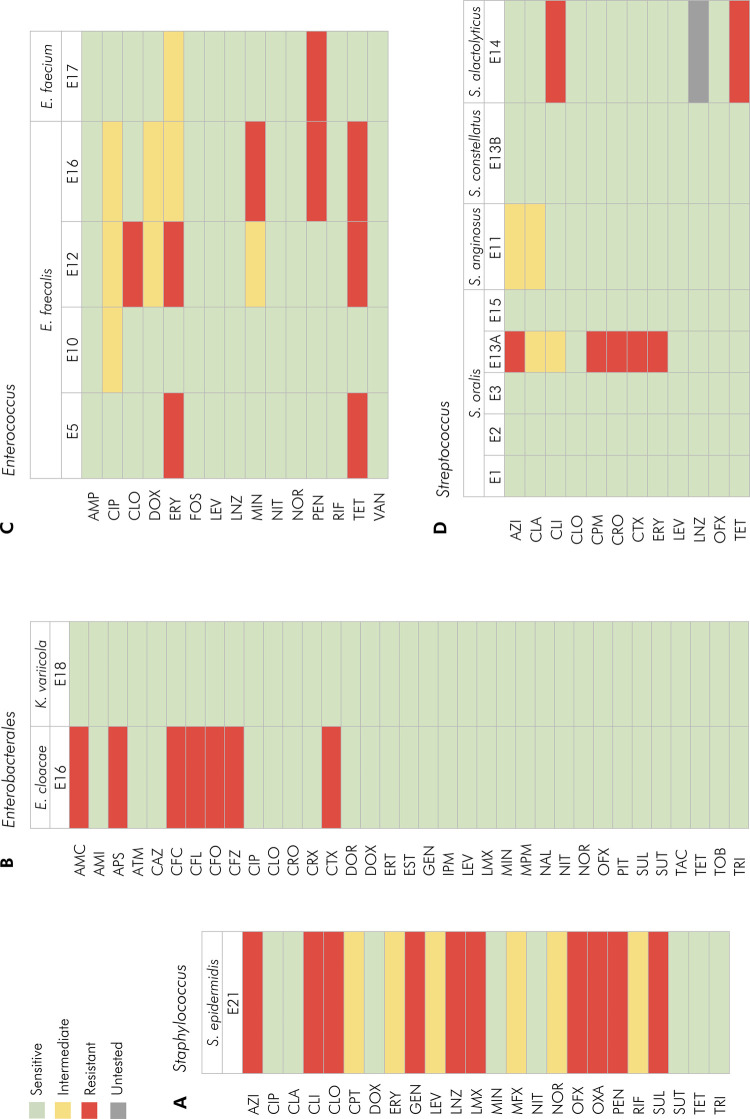
Antimicrobial susceptibility profile of the bacteria studied.

(A–D) Bacterial family or genus. All antimicrobials tested were amoxicillin-clavulanate (AMC), amikacin (AMI), ampicillin-sulbactam (APS), ampicillin (AMP), aztreonam (ATM), azithromycin (AZI), ceftazidime (CAZ), cefaclor (CFC), ceftaroline(CPT), cefoxitin (CFO), cefazolin (CFZ), cephalothin (CFL), ciprofloxacin (CIP), clarithromycin (CLA), clindamycin (CLI), chloramphenicol (CLO), cefepime (CPM), ceftriaxone (CRO), cefuroxime (CRX), cefotaxime (CTX), doripenem (DOR), doxycycline (DOX), ertapenem (ERT), erythromycin (ERY), streptomycin (EST), fosfomycin (FOS), gentamicin (GEN), imipenem (IPM), levofloxacin (LEV), linezolid (LNZ), lomefloxacin (LMX), minocycline (MIN), moxifloxacin (MFX), meropenem (MPM), nalidixic acid (NAL), nitrofurantoin (NIT), norfloxacin (NOR), ofloxacin (OFX), oxacillin (OXA), penicillin (PEN), piperacillin-tazobactam (PIT), rifampicin (RIF), sulphonamide (SUL), trimethoprim-sulfamethoxazole (SUT), ticarcillin-clavulanate (TAC), tetracycline (TET), tobramycin (TOB), trimethoprim (TRI), and vancomycin (VAN)

S. epidermidis, the isolate E21, was multidrug-resistant, showing no susceptibility to azithromycin, chloramphenicol, clindamycin, ceftaroline, erythromycin, gentamicin, levofloxacin, linezolid, lomefloxacin, moxifloxacin, norfloxacin, ofloxacin, oxacillin, penicillin, rifampicin, and sulfonamides (Figure).

Among the species of Enterobacterales species, the *K. variicola* isolate was susceptible to all antibiotics tested. On the other hand, E. cloacae showed no susceptibility to amoxicillin with clavulanic acid, ampicillin-sulbactam, cefaclor, cephalothin, cefoxitin, cefazolin, and cefotaxime (Figure). After isolation and identification, the isolate E20 *P. rettgeri* showed contamination in its culture, and it was impossible to perform the antimicrobial susceptibility test.

## Discussion

Studies focusing on characterizing bacteria involved in endodontic infections have grown in popularity to improve clinical practice and endodontic treatment.^4^ In this study, 15 of the 21 samples collected from primary endodontic infections showed bacterial growth, whereas six samples (E4, E6, E7, E8, E9, and E19) did not.

Once the endodontic infection is identified, the lack of microorganism growth can be explained by various factors, including the fact that the microorganisms causing the infection may be uncultivable in vitro or even strict anaerobic bacteria that do not grow under 5% CO_2_. Viruses, such as the herpes virus, can also be associated with endodontic infections.^18,20^ Although the sample collection criteria have been strictly controlled, failures during this process are possible. Approximately 55% of the bacteria associated with infected root canals of primary apical periodontitis are still uncultivable.^4,6^ In some cases, some of these in vitro non-cultivable bacteria may even predominate in endodontic infections, which helps to explain why culture may be ineffective in isolating and identifying bacteria present in these infections. In these cases, the lack of bacterial culture makes phenotypic studies of antimicrobial resistance impossible.^4^

Even though most endodontic infections are polymicrobial, with a predominance of strictly anaerobic bacteria, *E. faecalis*, a facultative anaerobic bacterium that causes persistent infections, has been found in infected RCS.^21^ Evidence suggests an association between *Enterococcus* spp., particularly *E. faecalis*, and primary and secondary endodontic infections.^22^ However, the present study found a higher prevalence of *E. faecalis*. Contrary to previous research, which found a low incidence of primary endodontic infection caused by this species.^2,23,24^ Recent studies have shown that primary and secondary endodontic infections can have diverse and similar microbiota, corroborating the results presented here.^25^ Furthermore, studies in Brazil have revealed the presence of *E. faecalis* in endodontic infections of deciduous teeth, highlighting the species’ importance in infections other than secondary RCS.^26^

According to research, the most common bacterial species in endodontic infections are S. constellatus and *S. mitis/oralis*, which are consistent with our findings.^27,28^ The present study included three *S. mitis/oralis* (E1, E13A, and E15 samples) and one S. constellatus (E13B). Both species were isolated from sample 13. These two species, *E. faecalis, E. faecium*, S. anginosus, S. epidermidis, S. alactolyticus, E. cloacae, *K. variicola*, and P. rettgeri were also found.

The S. mitis group includes several species, including *S. mitis* and *S. oralis*, among the most common oral colonizing microorganisms in humans.^28^ We describe these two associated species (*S. mitis/oralis*) in this study because the Vitek-2 automated system cannot differentiate them during identification. Because it is an opportunistic pathogen, both species are frequently isolated from root canal infections.^29^ They are facultative anaerobic species with great metabolic versatility, using a wider range of nutrients for metabolism, allowing them to grow in less favorable environments.^30^ These bacteria are known to be among the most important in forming dental biofilms. Furthermore, they can interact with Porphyromonas gingivalis, which is one of the primary causes of periodontal disease.^31^ Another frequent species in the study was S. anginosus, found in three samples (E2, E3, and E11). According to reports, in addition to *E. faecalis*, this species is described as one of the most common microorganisms in teeth with endodontic treatment failure.^4^

In this study, five *Enterococcus* spp. strains (four *E. faecalis* and one *E. faecium*) were isolated from 21 patients with primary endodontic infections (prevalence of 23.8%). *E. faecalis* has been associated with several oral diseases, including caries, periodontitis, peri-implantitis, and endodontic infections.^32^ This species is strongly linked to endodontic treatment failure due to its ability to form persistent biofilms in treated and untreated RCS, as well as its high resistance to endodontic drugs, including calcium hydroxide.^33^

Our research group published the *K. variicola* strain described in this study separately as the first worldwide report of this species causing primary endodontic infection. This species was initially identified as *K. pneumoniae* by the Vitek-2 system. After sequencing the complete genome, it was identified as *K. variicola*.^3^ This strain exhibited a hypermucoid phenotype, which can frequently spread from the site of infection to other areas.^3^ This is concerning in dentistry, particularly in endodontic treatments, because these infections can spread throughout the periapical region and progress to more severe cases, such as sinusitis and meningitis.

Antimicrobial susceptibility was found in the majority of bacterial isolates. Some isolates, however, were resistant to antimicrobials, such as erythromycin, tetracycline, and penicillin. Previous studies found a high incidence of bacterial resistance to tetracycline, an antibiotic still widely used in treating primary endodontic infections caused by *E. faecalis* isolates.^34^ Furthermore, other studies have found penicillin resistance in multidrug-resistant strains of *E. faecalis* isolated from persistent endodontic infections, with amoxicillin and clavulanate acid being the most effective treatment in these cases.^35,36,37^

This increased tetracycline resistance in endodontic isolates can be attributed to selective pressure caused by previous use of tetracyclines in dental practice, such as treating localized aggressive periodontitis and intracanal medication. In a 12-month longitudinal study, researchers examined the antimicrobial resistance profile of the subgingival microbiota after systemic and local use of tetracycline as an adjunct to periodontal therapy.^38^ In all cases where tetracycline was used, bacterial resistance increased, demonstrating that using tetracycline as an intracanal medication can promote the selection of resistant strains at the site of infection.^37^

In most endodontic procedures, antibiotics are prescribed in cases of acute apical infections or prophylactically in patients with clinical impairment. The microbial ecosystem of endodontic origin has recently sparked interest due to its potential as a reservoir of genetic elements endowed with resistance to various classes of antibiotics. In this context, studies focusing on identifying the prevalence of antimicrobial resistance in the endodontic microbiome are critical, providing endodontic professionals with financial incentives to adopt more effective therapeutic approaches.^38^

The lack of investigation of obligate anaerobes bacteria is one of the study’s limitations. Bacteria species investigated in this study included obligate aerobes, facultative anaerobes, aerotolerant anaerobes, and microaerophiles; however, obligate anaerobes bacterial that may be present in endodontic infections were not investigated. Another limitation is that uncultivable bacteria were not studied, even though they could have been studied using other techniques not used in the study, such as metagenomics. Fungi and viruses may also be present in endodontic infections, but this study did not include them.

## Conclusion

In the context of this study, it is possible to observe that the endodontic infections under investigation have a Gram-positive bacterial composition. The bacterial genera *Enterococcus* and Streptococcus are primarily responsible for such infections. In addition, less common occurrences of bacteria from the genera Enterobacter, Klebsiella, Staphylococcus, and Providencia have been identified.

The analysis of the obtained bacterial isolates revealed significant susceptibility to most antibiotic agents tested. It is worth noting, however, that some *Enterococcus* isolates showed resistance to the antibiotic erythromycin, ciprofloxacin, and tetracycline. An isolate of the species *Staphylococcus epidermidis* was identified as multiresistant, indicating a resistance profile to multiple antimicrobial agents. It is worth noting that five of the Streptococcus isolates showed patterns of nonsusceptibility to all antibiotics tested.

These findings suggest that most bacterial isolates in the endodontic infections studied are susceptible to antimicrobials. Multiresistant strains, on the other hand, have been identified, indicating the emergence of potential challenges in the control and treatment of endodontic infections caused by such bacteria. This necessitates a more comprehensive and strategic approach to antimicrobial agent use and clinical management of these situations. Nonetheless, the study is limited, and more extensive studies are needed to understand the emergence of antimicrobial resistance in endodontic infections.
